# Supplementary material for: Na+ Lattice Doping Induces Oxygen Vacancies to Achieve High Capacity and Mitigate Voltage Decay of Li-Rich Cathodes
Source: Int J Mol Sci. 2023 Apr 28;24(9):8035. doi: 10.3390/ijms24098035 (PMC10179001; doi:10.3390/ijms24098035)
Supplement: Supplementary file 1 [file ijms-24-08035-s001.zip › ijms-2343978-supplementary.pdf]

Na^+ Lattice Doping Induces Oxygen Vacancies to Achieve High Capacity and Mitigate Voltage Decay of Li-Rich Cathodes

Hengrui Qiu, Rui Zhang, Youxiang Zhang*

College of Chemistry and Molecular Sciences, Wuhan University, Wuhan 430072, China

E-mail: yxzhang04@whu.edu.cn

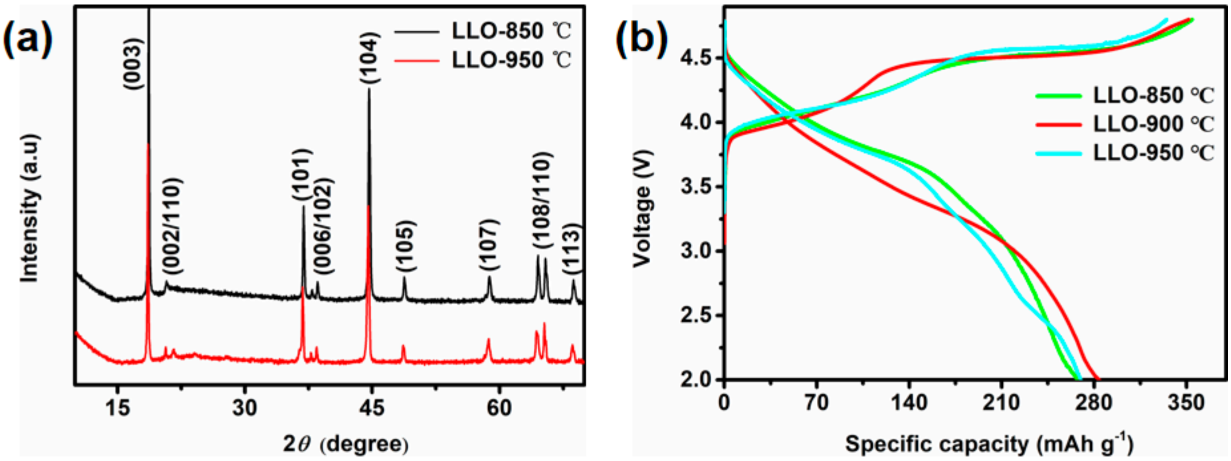

Figure S1. (a) XRD patterns of LLO-850 ℃, LLO-950 ℃ and (b) charge-discharge curves of LLO-850 ℃, LLO-950 ℃ and LLO-950 ℃ (i.e. 15-LLO-Na-OV).

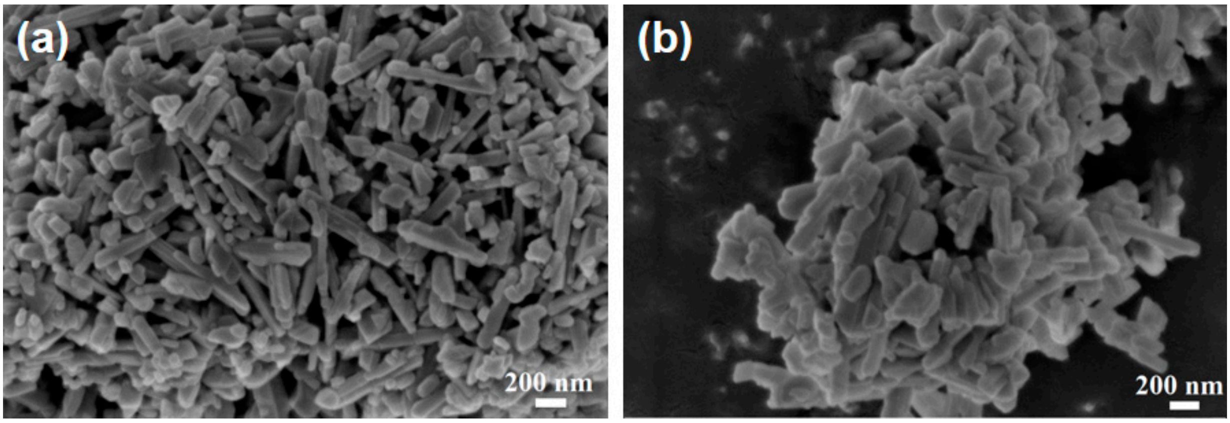

Figure S2. SEM images of (a) LLO-850 ℃ and (b) LLO-950 ℃.

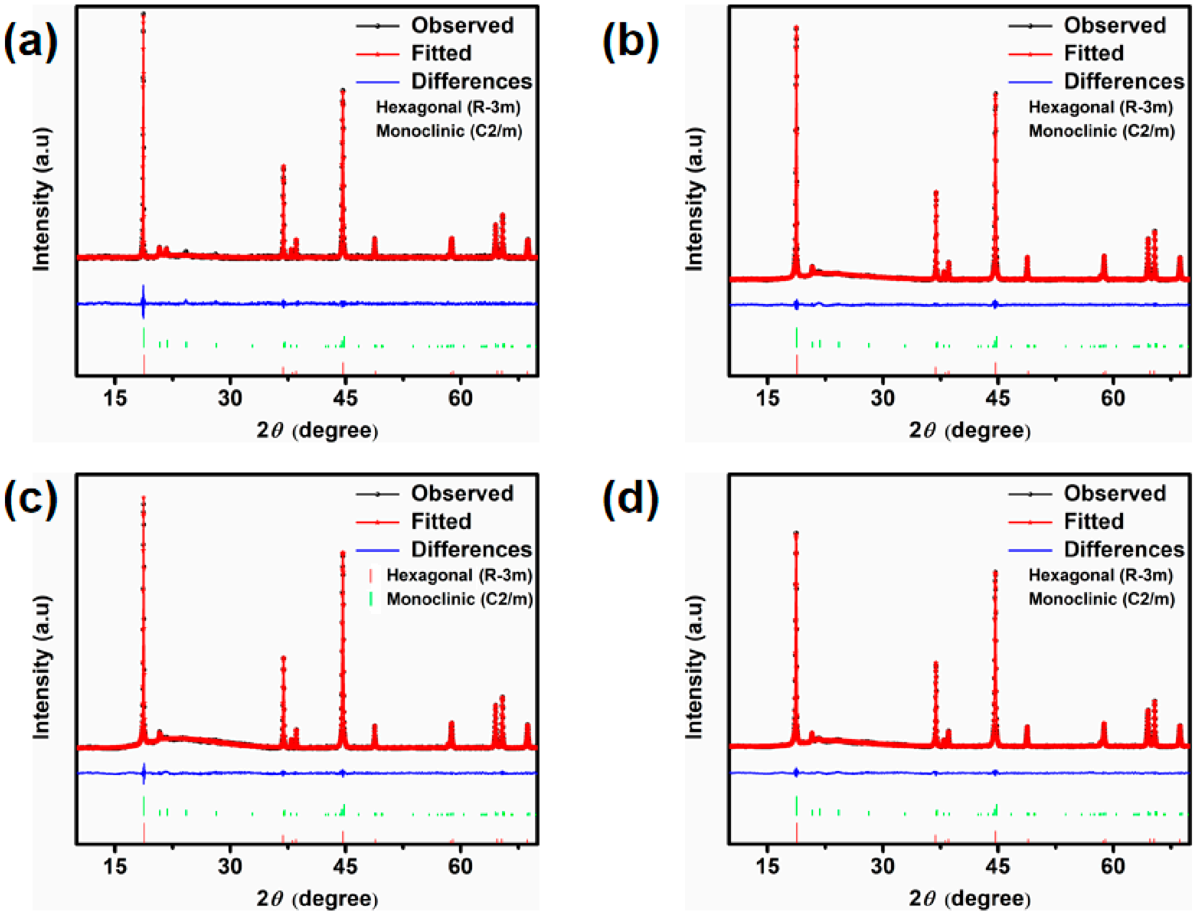

Figure S3. Rietveld refinement results of (a) Pristine-LLO, (b) 10-LLO-Na-OV, (c) 15-LLO-Na-OV and (d) 20-LLO-Na-OV.

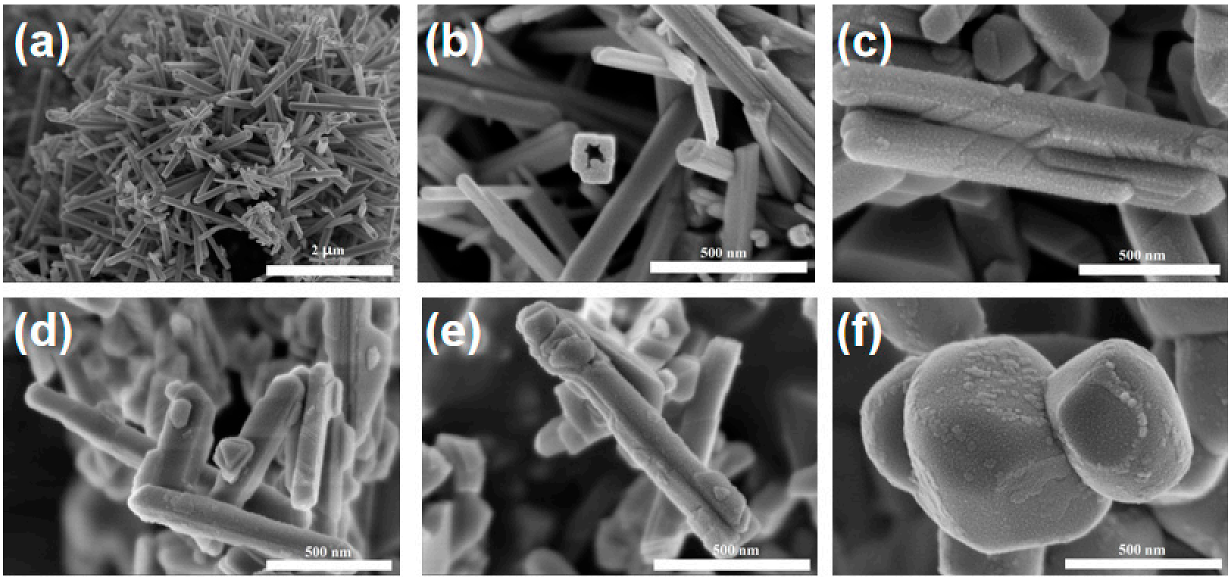

Figure S4. SEM images of (a and b) hollow MnO_2, (c) 10-LLO-Na-OV, (d) 15-LLO-Na-OV, (e) 20-LLO-Na-OV and (f) Pristine-LLO.

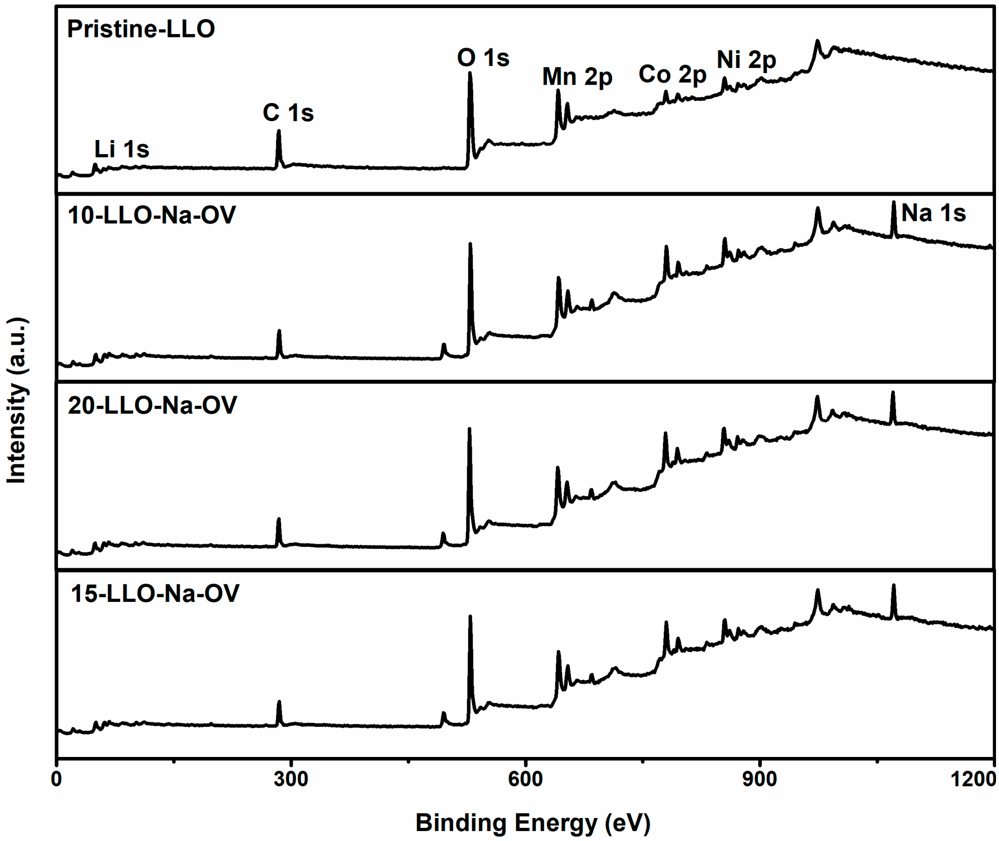

Figure S5. Survey spectrum of Pristine-LLO, 10-LLO-Na-OV, 15-LLO-Na-OV and 20-LLO-Na-OV.

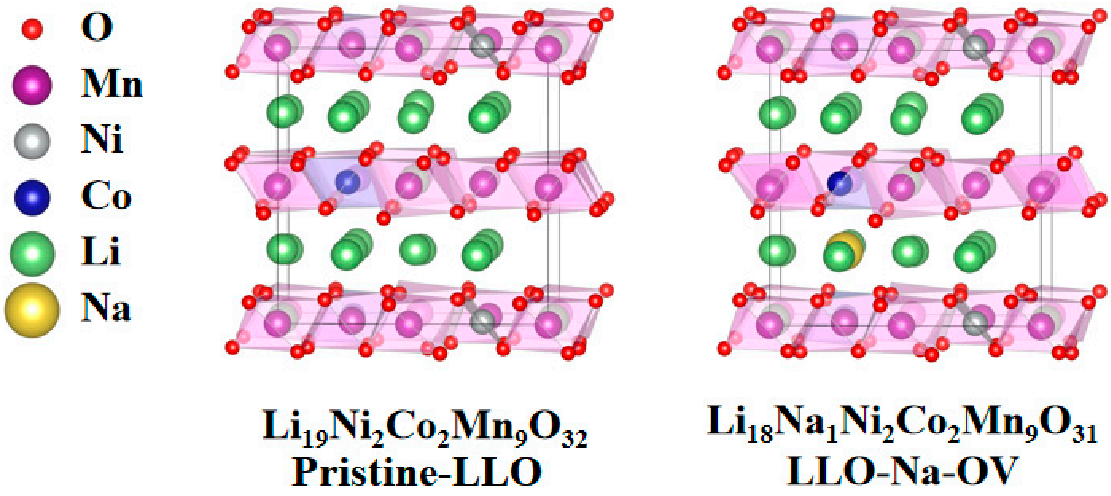

Figure S6. The crystal structures of Pristine-LLO and LLO-Na-OV models.

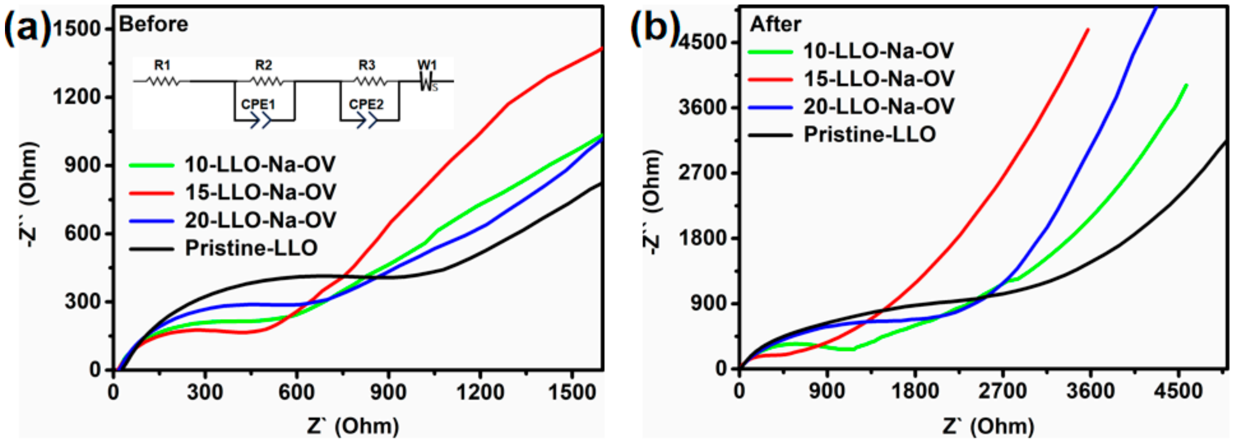

Figure S7. Nyquist plots of Pristine-LLO, 10-LLO-Na-OV, 15-LLO-Na-OV and 20-LLO-Na-OV: (a) Before cycling, (b) after cycling.

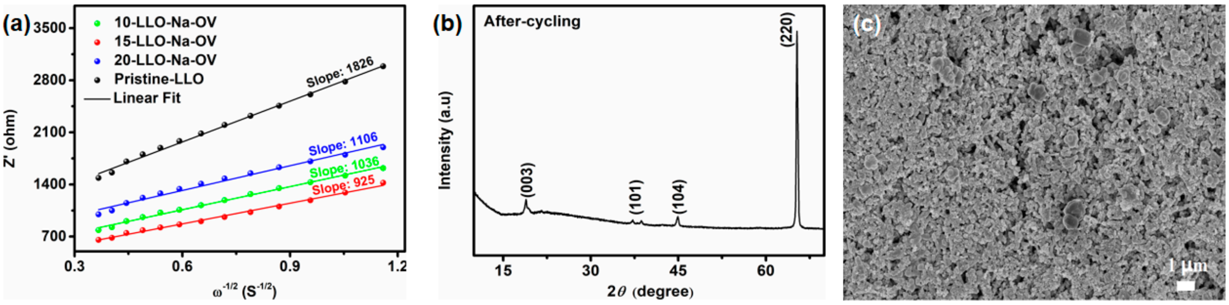

Figure S8. (a) Fitted curves of Z' with $\omega^{-1/2}$; (b) XRD pattern and (c) SEM image of 15-LLO-Na-OV after cycling.

Table S1. The chemical composition results of ICP analysis for Pristine-LLO, 10-LLO-Na-OV, 15-LLO-Na-OV and 20-LLO-Na-OV samples.

Samples/parameters	Li	Ni	Co	Mn	Na
Pristine-LLO	1.2032	0.1311	0.1312	0.54	0
10-LLO-Na-OV	1.2039	0.1314	0.1315	0.54	0.0039
15-LLO-Na-OV	1.2052	0.1318	0.1316	0.54	0.0080
20-LLO-Na-OV	1.2064	0.1319	0.1307	0.54	0.0112

Table S2. Rietveld refinements of the XRD data of Pristine-LLO, 10-LLO-Na-OV, 15-LLO-Na-OV and 20-LLO-Na-OV samples.

Samples/parameters	a (Å)	c (Å)	c/a	Rwp (%)	Rp (%)
Pristine-LLO	2.8502	14.2124	4.9865	5.95	2.92
10-LLO-Na-OV	2.8501	14.2187	4.9888	5.13	3.45
15-LLO-Na-OV	2.8497	14.2215	4.9905	4.78	2.94
20-LLO-Na-OV	2.8515	14.2306	4.9906	6.27	3.89

Table S3. The discharge capacity and initial coulombic efficiency of Pristine-LLO, 10-LLO-Na-OV, 15-LLO-Na-OV and 20-LLO-Na-OV samples.

Samples/parameters	Pristine-LLO	10-LLO-Na-OV	15-LLO-Na-OV	20-LLO-Na-OV
Discharge capacity at 0.1 C (mAh g^{-1})	219	269	282	281
Initial coulombic efficiency (%)	71.67	76.83	80.76	74.24

Table S4. The comparison of cycling performances and initial capacity between this work and previously reported ones.

Samples/parameters	1 C (mA g^{-1})	capacity retention	initial capacity	Ref.
Pre-generated oxygen vacancies and oxygen-deficient phase $Li_{1.2}Mn_{0.6}Ni_{0.2}O_2$	200	77.2% 200 cycles at 1 C	271.4 mAh g^{-1} at 0.1 C	43
Surface protective coating $Li_{1.2}Ni_{0.13}Co_{0.13}Mn_{0.54}O_2$	250	92.4% 250 cycles at 1 C	228.5 mAh g^{-1} at 0.1 C	44
Na doped Ni excess $Li_{1.2-x}Na_xMn_{0.52}Co_{0.08}Ni_{0.20}O_2$	200	97.83% 200 cycles at 0.5 C	184.0 mAh g^{-1} at 0.5 C	45
Sodium doping $Li_{1.2}Ni_{0.13}Co_{0.13}Mn_{0.54}O_2$	200	85.4% 100 cycles at 1 C	265.2 mAh g^{-1} at 0.1 C	46
Na_3PO_4 as a dopant $Li_{1.2}Ni_{0.13}Co_{0.13}Mn_{0.54}O_2$	250	93.80% 100 cycles at 1 C	255.7 mAh g^{-1} at 0.1 C	47
This work	250	90.02% 150 cycles at 1 C	282.0 mAh g^{-1} at 0.1 C	/

Table S5. The fitted Rs, Rct values before and after cycling and the corresponding σ and D_{Li}^+ of Pristine-LLO, 10-LLO-Na-OV, 15-LLO-Na-OV and 20-LLO-Na-OV samples.

Samples/parameters	Pristine-LLO	10-LLO-Na-OV	15-LLO-Na-OV	20-LLO-Na-OV
Rs-before (Ω)	20.35	15.36	14.25	17.15
Rct-before (Ω)	945.54	565.10	528.24	860.3
Rs-after (Ω)	38.69	23.54	19.89	26.71
Rct-after (Ω)	1899.64	903.8	556.30	1554
σ ($Ω·cm^2·s^{-1/2}$)	1826	1036	925	1106
D_{Li}^+ ($cm^2·s^{-1}$)	$1.61*10^{-16}$	$5.00*10^{-16}$	$6.28*10^{-16}$	$4.39*10^{-16}$

Table S6. The detailed deconvolution parameters of Pristine-LLO, 10-LLO-Na-OV, 15-LLO-Na-OV and 20-LLO-Na-OV samples.

Parameters (cm^{-1})/Samples	Pristine-LLO	10-LLO-Na-OV	15-LLO-Na-OV	20-LLO-Na-OV
A_{1g}-before	589.12	589.12	591.60	594.09
A_{1g}-after	607.86	595.70	617.51	612.68
E_g-before	470.20	472.62	472.74	473.51
E_g-after	501.07	476.81	499.46	486.40
I_R-before	590.87	588.83	591.60	594.09
I_R-after	605.37	589.78	614.29	598.79
I_S-before	645.41	644.20	646.15	647.02
I_S-after	635.51	631.52	627.31	629.80
FWHM-I_R-before	55.95	57.23	53.52	55.62
FWHM-I_R-after	33.32	59.11	77.83	77.87
FWHM-I_S-before	55.97	57.33	53.53	52.63
FWHM-I_S-after	72.02	60.11	77.84	70.40
